# Development and validation of a triglyceride-glucose integrated nomogram for acute kidney injury prediction in acute myocardial infarction patients: a multicenter database study

**DOI:** 10.3389/fcvm.2025.1620664

**Published:** 2025-09-02

**Authors:** Xinling Zhang, Ranran Ding, Guangming Yang, Yan Jiang, Yaping Feng, Feng Qu, Yuling Qiao, Qiang Meng

**Affiliations:** ^1^ Department of Healthcare Ward, Jining No. 1 People’s Hospital, Jining, Shandong, China; ^2^ Department of Intensive Care Unit, Jining No. 1 People’s Hospital, Jining, Shandong, China

**Keywords:** triglyceride-glucose index, acute myocardial infarction, acute kidney injury, nomogram, insulin resistance

## Abstract

**Background:**

Acute kidney injury (AKI) is a life-threatening complication in patients with acute myocardial infarction (AMI), leading to increased morbidity and mortality. Early prediction of high-risk patients remains a clinical challenge.

**Methods:**

We developed and validated a predictive model for AKI using data from two large critical care databases: MIMIC-IV (*n* = 1,227) and eICU (*n* = 1,954). Least absolute shrinkage and selection operator (LASSO) regression and multivariable logistic regression were applied to identify independent predictors. A nomogram was constructed incorporating the triglyceride-glucose (TyG) index and clinical variables.

**Results:**

Seven predictors were included in the final model: TyG index, blood urea nitrogen (BUN), SOFA score, age, serum sodium, serum albumin and systolic blood pressure (SBP). The model demonstrated excellent discrimination with area under the curve (AUC) values of 0.85 in the training cohort, 0.83 in the internal validation cohort and 0.81 in the external validation cohort. Decision curve analysis showed clinical usefulness across a wide range of risk thresholds (22%–45%). The TyG index was independently associated with increased AKI risk (odds ratio 1.31; 95% CI: 1.07–1.60). The model also showed improved risk reclassification (net reclassification index: 0.22; *p* < 0.001).

**Conclusion:**

The TyG-based nomogram provides a practical and accurate tool for early prediction of AKI in AMI patients. By integrating metabolic, hemodynamic, and organ dysfunction markers, this model enables multidimensional risk stratification and may support timely preventive strategies in the ICU setting.

## 1 Introduction

Despite significant advancements in pharmacological and interventional therapies over the past decades, acute myocardial infarction (AMI) remains a leading cause of morbidity and mortality worldwide (1). Acute kidney injury (AKI) is a common and serious complication in patients with acute myocardial infarction (AMI), with an incidence reaching 59% in hospitalized patients (2). The development of AKI in patients with AMI not only prolongs hospitalization and increases healthcare costs, but also independently associated with higher short- and long-term mortality (3, 4). Moreover, AKI may cause lasting renal dysfunction and even progress to chronic kidney disease, thereby increasing the long-term healthcare burden, even in patients who recover from the initial event (5). However, the current diagnostic criteria for AKI often require either baseline serum creatinine levels or urine output monitoring over a minimum of 6 h, both of which may delay diagnosis in acute care settings. As a result, patients frequently miss the optimal window for therapeutic intervention. Therefore, early identification of high-risk patients and implementation of preventive interventions are crucial for reducing AKI incidence and improving clinical outcomes in AMI patients. The development of predictive models based on novel biomarkers or machine learning algorithms represents a critical breakthrough for early AKI identification in AMI patients. Although the underlying pathophysiological mechanisms underlying AKI in patients with acute myocardial infarction (AMI) are not yet fully elucidated, emerging evidence suggests that the triglyceride-glucose (TyG) index may offer new perspectives for AKI prediction.

TyG index serves as a clinically validated surrogate marker for insulin resistance (IR), which is pathophysiologically defined as a state of diminished insulin sensitivity in key metabolic tissues including the liver, skeletal muscle, and adipose tissue, ultimately leading to impaired glucose uptake and utilization (6). Beyond its established role in diabetes prediction where it outperforms conventional blood glucose measurements (7), this metabolic disturbance exerts broader systemic effects by disrupting homeostatic balance. Specifically, IR contributes to cardiovascular and renal pathophysiology through multiple interconnected mechanisms such as chronic low-grade inflammation, endothelial cell dysfunction, and dysregulated lipid metabolism (8, 9). These observations provide a mechanistic foundation for investigating the TyG index as a potential predictor of AKI risk in the AMI population. In recent years, the TyG index has garnered significant attention due to its outstanding predictive capabilities, particularly in identifying risks associated with metabolic and cardiovascular conditions, as well as its ability to stratify mortality risk in critically ill patients across diverse clinical scenarios (10–12). However, despite the growing interest in the TyG index, there is still a lack of comprehensive, clinically applicable tools for predicting AKI in AMI patients. Insulin resistance, as quantified by the TyG index, is influenced by multiple interacting factors including metabolic abnormalities such as dyslipidemia, hemodynamic alterations like renal hypoperfusion, and systemic inflammation. This complex pathophysiology is supported by recent mechanistic studies (13). Although previous studies have evaluated effect modification by hypertension, diabetes, age, and sex through stratified analyses (10), residual confounding may persist due to unadjusted factors such as medication use and comorbidities. These limitations highlight the need for broader validation across diverse clinical populations. Furthermore, although a TyG index value exceeding 9.2 has demonstrated prognostic significance in observational studies (14), this dichotomous cutoff has limited clinical utility. In practice, clinicians require probabilistic risk estimation rather than binary classification, as the same TyG value may correspond to different absolute AKI risks depending on additional factors such as left ventricular function and baseline kidney status. Therefore, there is a pressing need to integrate multiple clinical factors with biomarkers like the TyG index to develop a more accurate and practical predictive model.

This study aims to develop and validate a nomogram for predicting AKI in acute myocardial infarction patients using the TyG index in combination with other clinical variables. By incorporating both easily accessible clinical factors and the TyG index, this model could provide clinicians with a valuable tool for early risk stratification, allowing for timely interventions and potentially improving patient outcomes. Using data from the MIMIC-IV database of critically ill patients, we will employ advanced statistical techniques to develop and validate the nomogram, ensuring its robustness and clinical applicability. The development of a TyG-enhanced nomogram could improve clinical practice by providing a more accurate and user-friendly tool for early AKI prediction, ultimately reducing the incidence of AKI and improving patient outcomes.

## 2 Methods

### 2.1 Data source

We utilized data from two large, open-source databases: Medical Information Mart for Intensive Care IV (MIMIC-IV) version 3.1 (15) and eICU-Collaborative Research database (eICU-CRD) version 2.0 (16). MIMIC-IV 3.1 extends its predecessor (version 2.0) by including ICU admissions from 2020 to 2022, expanding the total patient population to over 94,000. Maintained by the Beth Israel Deaconess Medical Center, this database has become a widely utilized resource in critical care research (17). The eICU Collaborative Research Database is a multi-center database comprising deidentified health data associated with over 200,000 admissions to ICUs across the United States between 2014 and 2015. The author (Qiang Meng) completed the National Institutes of Health (NIH) web-based training course, “Protecting Human Research Participants” (certification number: 56251014), as required for data access. Ethical approval was obtained from the Institutional Review Boards of the Massachusetts Institute of Technology (Cambridge, MA, USA) and the Beth Israel Deaconess Medical Center, with a waiver of informed consent due to the retrospective nature of the study and the use of deidentified data.

### 2.2 Patient selection

Patients were included if they met the following criteria: (1) Admission to the intensive care unit (ICU) with a primary diagnosis of acute myocardial infarction (AMI); (2) Availability of fasting triglyceride and glucose measurements within 24 h of ICU admission for TyG index calculation, using the formula: $\mathrm{TyG}\;\mathrm{index}=\mathrm{Ln}\frac{\mathrm{TG}(\mathrm{mg}/\mathrm{dL})*\mathrm{FBG}(\mathrm{mg}/\mathrm{dL})}{2}$; (3) Age ≥18 years; (4) ICU length of stay ≥24 h. Patients were excluded if they met any of the following criteria: (1) End-stage renal disease (ESRD) or chronic dialysis; (2) Missing data required for TyG index calculation or AKI diagnosis. (3) Multiple ICU admissions (only the first ICU stay was included).

### 2.3 Data extraction and definition

Data were extracted using Structured Query Language (SQL) in Navicat Premium (version 15.0.12). Patients with acute myocardial infarction (AMI) were identified from the database using ICD-9 (International Classification of Diseases, 9th Revision; codes 41000–41092) and ICD-10 (codes I21–I219). Data extracted within the first 24 h of ICU admission included demographic information, clinical parameters, vital signs, laboratory parameters, severity scores, comorbidities, and therapies. Demographic information comprised gender, age, body mass index (BMI), and race. Clinical parameters included length of stay (LOS) in the intensive care unit (ICU) and LOS in the hospital. Vital signs consisted of heart rate (HR), systolic blood pressure (SBP), diastolic blood pressure (DBP), and respiratory rate (RR). Laboratory parameters encompassed white blood cell count (WBC), red blood cell count (RBC), platelet count, hemoglobin level, serum creatinine (SCr), blood urea nitrogen (BUN), creatine kinase-MB (CK-MB), albumin, fasting blood glucose (FBG), serum sodium, serum potassium, calcium, chloride, bicarbonate, total triglyceride (TG), international normalized ratio (INR), partial thromboplastin time (PTT), alanine aminotransferase (ALT), aspartate aminotransferase (AST), and alkaline phosphatase (ALP). Severity at admission was assessed using the Simplified Acute Physiological Score II (SAPS II), Systemic Inflammatory Response Syndrome (SIRS) score, Acute Physiology Score III (APS III), and the Sequential Organ Failure Assessment (SOFA) score. Comorbidities included chronic kidney disease (CKD), chronic pulmonary disease, liver disease, diabetes, and hypertension. Therapies comprised percutaneous coronary intervention (PCI), coronary artery bypass grafting (CABG), and renal replacement therapy (RRT). To address missing data, a systematic and stratified approach was implemented based on the extent of missingness. For variables with less than 20% missing values, the missForest algorithm in R software was utilized for multiple imputation. For variables with missing values in the range of 20%–50%, specifically CK-MB, a categorical transformation strategy was adopted, converting these variables into dummy indicators to avoid the potential biases introduced by direct imputation (18). Variables with more than 50% missing data, including B-type natriuretic peptide (BNP), C-reactive protein (CRP), and cardiac troponin I (cTNI) were excluded from the analysis to ensure the reliability and validity of the results. This approach not only maintains dataset integrity but also reduces potential bias, thereby strengthening the robustness of the findings.

To evaluate potential bias introduced by data exclusion, we conducted two additional analyses. First, to assess the impact of excluding patients with missing triglyceride or glucose values, we compared baseline characteristics between the final study cohort and those excluded after removal of duplicate records. Second, to examine the influence of early in-hospital mortality on model performance, we performed a sensitivity analysis by excluding patients who died before the onset of AKI, and reassessed model discrimination in the adjusted cohort.

The endpoint was AKI developing within 7 days following ICU admission. The diagnosis of AKI was based on the latest international clinical practice guidelines for AKI (19), and accordance to any of the following three criteria: (1) creatinine rose ≥ 0.3 mg/dl within 0 h; (2) serum creatinine elevation ≥50% above baseline within 7 days; and (3) urine output < 0.5 ml/kg/h over 6 h.

### 2.4 Model development and validation

The dataset extracted from the MIMIC-IV database was randomly partitioned into training and internal validation cohorts using a 7:3 ratio. Specifically, 70% of the data were allocated for model training, while the remaining 30% were reserved for internal validation. This stratified randomization approach ensured balanced representation of clinical characteristics across both subsets. The eICU-CRD served as an external validation cohort, enabling assessment of model generalizability across diverse healthcare settings.

Feature selection was conducted through a sequential analytical approach. Prior to modeling, all continuous variables were standardized to ensure comparability. Least Absolute Shrinkage and Selection Operator (LASSO) regression with 5-fold cross-validation was then applied for preliminary feature selection by shrinking the coefficients of less relevant variables to zero, thereby addressing potential multicollinearity and reducing dimensionality. Subsequently, univariate logistic regression was conducted on the LASSO-selected features, and variables with *p* < 0.05 were included in a multivariate logistic regression to identify independent predictors. Finally, multivariate logistic regression was performed to identify independent predictors, with statistically significant variables (*p* < 0.05) assessed for their effect size using odds ratios (OR) and corresponding 95% confidence intervals.

The final selected variables were utilized to construct a predictive nomogram using the “rms” package in R software. To evaluate the model's discriminative performance, receiver operating characteristic (ROC) curves were generated and the area under the curve (AUC) was calculated. Additional performance metrics—including F1 score, recall, precision, and accuracy—were reported for a more comprehensive assessment. To further evaluate the added predictive value of the TyG index, two logistic regression models were developed based on the selected features: a full model incorporating the TyG index (TyG model), and a nested model excluding the TyG (non-TyG model). These models were applied to the training, internal validation, and external validation cohorts for performance comparison. Calibration curves were generated using the “rms” package with 1,000 bootstrap resamples to assess the agreement between predicted probabilities and observed outcomes. To quantify the improvement in risk prediction brought by the TyG index, net reclassification improvement (NRI) and integrated discrimination improvement (IDI) were calculated. Finally, the clinical utility of the model was assessed using decision curve analysis (DCA), which compares the net benefit of each model across a range of threshold probabilities (0–1, increment = 0.2), relative to the default “treat-all” and “treat-none” strategies.

To explore potential nonlinear relationships between the TyG index and the outcome in the final multivariate model, restricted cubic spline (RCS) analysis was conducted *post hoc* using the rms package in R software. The analysis focused on TyG without additional adjustment for other covariates, as its functional form within the final model was of primary interest. Four equally spaced knots were set to divide the TyG distribution into four equal parts. The linearity assumption was evaluated using a likelihood ratio test comparing models with linear and spline terms.

### 2.5 Statistical analysis

Descriptive statistics were computed for all categorical and continuous variables. Continuous variables were expressed as mean ± standard deviation (SD) or median (interquartile range, IQR), and categorical variables were expressed as frequencies (percentages). The chi-squared test or Fisher's exact test was used to compare categorical variables, and Student's t-test or Mann–Whitney U test was applied for continuous variables, as appropriate. All statistical analyses were performed using R software (v 4.2.0), with a two-sided *p* value < 0.05 considered statistically significant.

## 3 Results

### 3.1 Study population

**Figure 1:**
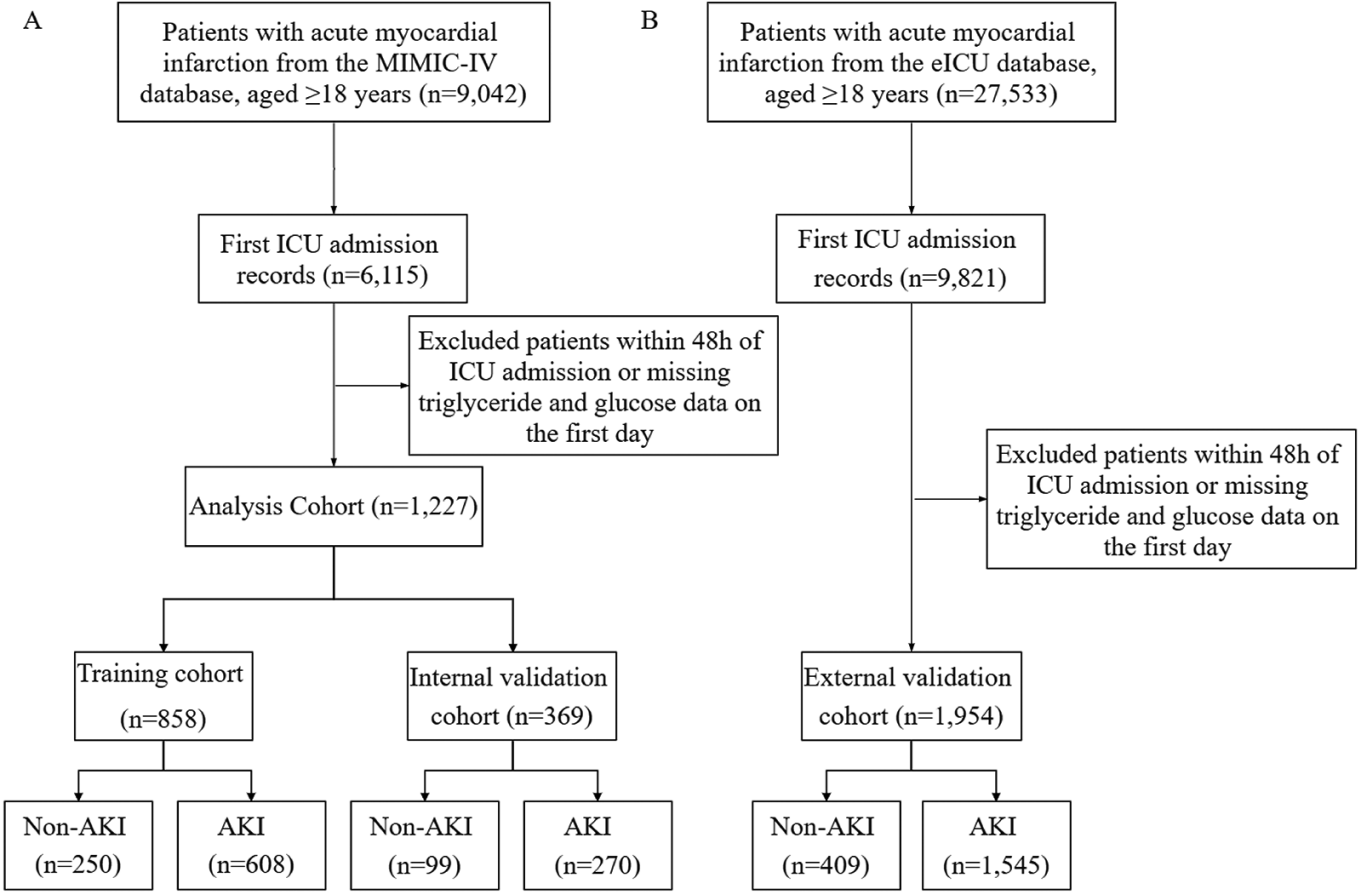
Study population flowchart and cohort selection. **(A)** Patients select from MIMIC-IV databases for model training and internal validation sets. **(B)** Patients select from e-ICU database for external validation set. MIMIC-IV, medical information mart for intensive care-IV; eICU-CRD, eICU-Collaborative Research database.

A total of 9,042 patients diagnosed with acute myocardial infarction (AMI) and admitted to the ICU were initially identified from the MIMIC-IV database. After excluding duplicate ICU admissions, patients aged ≤18 years, those with ICU stays <48 h, and cases lacking triglyceride or fasting blood glucose data, 1,227 eligible patients were included in the final cohort. Among them, 878 (71.5%) patients developed acute kidney injury (AKI). The MIMIC-IV dataset was randomly divided into a training cohort (*n* = 858) and an internal validation cohort (*n* = 369), while the eICU database served as an external validation cohort evaluate the generalizability of the model, where 1,545 (79.1%) patients experienced AKI (Figure 1). Baseline characteristics were compared between the training cohort and internal validation cohort (Table 1). The baseline characteristics of the external validation cohort are presented in Supplementary Table S1.

**Table 1:** Baseline characteristics in the training and internal validation cohort.

Variables	Training cohort				Internal validation cohort			
	Total (*n* = 858)	Non-AKI (*n* = 250)	AKI (*n* = 608)	*p*-value	Total (*n* = 369)	Non-AKI (*n* = 99)	AKI (*n* = 270)	*p*-value
Demographic								
Age, year	67.21 ± 12.32	66.34 ± 12.43	67.57 ± 12.27	0.186	67.18 ± 13.26	65.18 ± 12.92	67.91 ± 13.34	0.076
Gender, *n* (%)				0.201				0.596
Female	262 (31)	68 (27)	194 (32)		114 (31)	28 (28)	86 (32)	
Male	596 (69)	182 (73)	414 (68)		255 (69)	71 (72)	184 (68)	
BMI, kg/m^2^	29.35 ± 6.92	28.24 ± 5.96	29.81 ± 7.23	0.001	29.42 ± 6.33	28.87 ± 5.46		0.266
Race, *n* (%)				0.06				0.655
White	483 (56)	147 (59)	336 (55)		221 (60)	57 (58)	164 (61)	
Black	47 (5)	13 (5)	34 (6)		21 (6)	8 (8)	13 (5)	
Asian	32 (4)	15 (6)	17 (3)		5 (1)	1 (1)	4 (1)	
Other	296 (34)	75 (30)	221 (36)		122 (33)	33 (33)	89 (33)	
Vital sign								
Heart rate, b/s	82.87 ± 14.83	80.33 ± 14.04	83.92 ± 15.02	<0.001	82.09 ± 13.55	82.52 ± 13.71	81.94 ± 13.52	0.721
SBP, mmHg	113.98 ± 15.35	117.62 ± 15.28	112.49 ± 15.14	<0.001	113.99 ± 15.05	115.95 ± 15.88	113.27 ± 14.7	0.144
DBP, mmHg	64.25 ± 10.87	66.99 ± 11.06	63.12 ± 10.59	<0.001	63.31 ± 11.22	66.13 ± 10.51	62.28 ± 11.31	0.003
Laboratory tests								
Tg, mg/dl	115 (84, 168.75)	108.5 (80, 160.75)	117 (85, 171)	0.031	114 (82, 169)	114 (76, 169.5)	115.5 (83.5, 168.5)	0.697
Glucose, mg/dl	139 (112, 190)	126 (107, 162.75)	146 (115, 198)	<0.001	141 (114, 203)	132 (106, 164)	144 (117, 210)	0.013
TyG, (mg/dl)^2^	9.12 ± 0.77	8.95 ± 0.69	9.2 ± 0.79	<0.001	9.13 ± 0.79	9 ± 0.77	9.18 ± 0.79	0.059
Creatinine, mg/dl	1.0 (0.8, 1.5)	0.9 (0.8, 1.2)	1.1 (0.8, 1.7)	<0.001	1.1 (0.8, 1.4)	0.9 (0.8, 1.3)	1.1 (0.9, 1.5)	0.024
BUN, mg/dl	19 (14, 30)	17 (13, 23)	20 (15, 32)	<0.001	20 (15, 30)	18 (13.5, 30)	20 (15.25, 30)	0.09
Albumin, g/dl	3.48 ± 0.6	3.73 ± 0.44	3.38 ± 0.63	<0.001	3.49 ± 0.58	3.7 ± 0.47	3.41 ± 0.6	< 0.001
INR, s	1.2 (1.1, 1.4)	1.2 (1.1, 1.4)	1.2 (1.1, 1.4)	0.027	1.2 (1.1, 1.5)	1.2 (1.1, 1.4)	1.3 (1.1, 1.5)	0.134
PT, s	13.4 (12.3, 15.5)	13.1 (12.22, 14.78)	13.55 (12.4, 15.7)	0.013	13.5 (12.5, 16.1)	13.3 (12.35, 15)	13.75 (12.5, 16.38)	0.101
PTT, s	37.4 (29.42, 62.27)	38.2 (30, 60.95)	37.15 (29, 62.73)	0.526	37.2 (29, 61.5)	37.2 (29.75, 62.6)	37.15 (28.9, 61.45)	0.753
ALT, IU/L	33 (22, 66)	30 (22, 55)	34 (22, 76.25)	0.01	33 (22, 65)	30 (23.5, 48)	34 (22, 89)	0.066
AST, IU/L	62 (33, 147)	56 (32.25, 103.75)	62 (33, 174)	0.047	65 (32, 134)	54 (32, 98)	72 (33, 164.75)	0.011
ALP, IU/L	76 (63, 99.75)	74 (60, 85)	78 (64, 106.25)	0.002	76 (63, 99)	76 (62, 89.5)	77.5 (64, 104.75)	0.117
CK-MB, *n* (%)				0.046				0.348
<3	40 (5)	8 (3)	32 (5)		19 (5)	4 (4)	15 (6)	
>18	326 (38)	108 (43)	218 (36)		150 (41)	36 (36)	114 (42)	
3–6	72 (8)	12 (5)	60 (10)		39 (11)	12 (12)	27 (10)	
6–18	129 (15)	39 (16)	90 (15)		44 (12)	17 (17)	27 (10)	
Missing	291 (34)	83 (33)	208 (34)		117 (32)	30 (30)	87 (32)	
Sodium, mEq/L	138 (135, 140)	137.5 (135,139)	138 (135, 141)	0.015	138 (136, 140)	138 (135, 139)	138 (136, 140)	0.106
Calcium, mEq/L	8.52 ± 0.8	8.67 ± 0.72	8.46 ± 0.82	<0.001	8.5 ± 1.03	8.64 ± 0.77	8.44 ± 1.11	0.056
Chloride, mEq/L	102.97 ± 5.46	102.81 ± 4.74	103.03 ± 5.74	0.564	102.49 ± 5.87	101.99 ± 5.41	102.67 ± 6.02	0.304
Potassium, mEq/L	4.31 ± 0.66	4.23 ± 0.61	4.34 ± 0.67	0.026	4.3 ± 0.71	4.26 ± 0.72	4.31 ± 0.7	0.549
Bicarbonate, mmol/L	22.06 ± 4.29	22.65 ± 3.34	21.82 ± 4.6	0.003	22.44 ± 4.65	22.9 ± 3.87	22.27 ± 4.9	0.198
WBC, K/μl	13.17 ± 5.79	12.18 ± 5.86	13.59 ± 5.72	0.001	13.57 ± 15.24	11.78 ± 3.91	14.22 ± 17.62	0.033
RBC, K/μl	3.79 ± 0.73	3.9 ± 0.7	3.75 ± 0.74	0.004	3.79 ± 0.75	3.97 ± 0.77	3.73 ± 0.73	0.008
Hemoglobin, g/dl	11.39 ± 2.17	11.66 ± 2.09	11.28 ± 2.19	0.017	11.4 ± 2.22	11.79 ± 2.27	11.26 ± 2.19	0.044
Platelet, K/μl	192.4 (149.57, 245.75)	204.8 (171.62, 253.7)	186.5 (144.5, 241.77)	<0.001	199 (149, 246.5)	209 (171.5, 264.5)	194.6 (145.25, 237.4)	0.032
Severity scores								
SAPS II	38.17 ± 15.15	30.58 ± 11.02	41.29 ± 15.51	<0.001	37.14 ± 14.78	30.17 ± 12.67	39.7 ± 14.7	< 0.001
SOFA	4.9 ± 3.66	2.86 ± 2.67	5.74 ± 3.68	<0.001	4.8 ± 3.64	3 ± 2.88	5.47 ± 3.67	< 0.001
SIRS	2.61 ± 0.94	2.33 ± 0.99	2.73 ± 0.89	<0.001	2.6 6 ± 0.94	2.42 ± 0.99	2.75 ± 0.91	0.003
APS III	44.85 ± 23.36	33.7 ± 15.86	49.43 ± 24.4	<0.001	45.47 ± 23.83	36.74 ± 19.18	48.67 ± 24.59	< 0.001
OASIS	32.13 ± 9.21	27.05 ± 6.99	34.22 ± 9.21	<0.001	32.37 ± 8.9	28.02 ± 7.83	33.96 ± 8.74	<0.001
LODS	4.99 ± 3.36	2.99 ± 2.27	5.82 ± 3.39	<0.001	4.96 ± 3.18	3.3 ± 2.41	5.56 ± 3.22	<0.001
Comorbidities								
CKD, *n* (%)	229 (27)	54 (22)	175 (29)	0.038	102 (28)	22 (22)	80 (30)	0.201
COPD, *n* (%)	88 (10)	16 (6)	72 (12)	0.024	48 (13)	10 (10)	38 (14)	0.406
Hypertension, *n* (%)	458 (53)	138 (55)	320 (53)	0.542	187 (51)	53 (54)	134 (50)	0.584
Diabetes, *n* (%)	270 (31)	65 (26)	205 (34)	0.033	135 (37)	37 (37)	98 (36)	0.945
Treatment measures								
PCI, *n* (%)	65 (8)	24 (10)	41 (7)	0.195	21 (6)	5 (5)	16 (6)	0.946
CABG, *n* (%)	232 (27)	64 (26)	168 (28)	0.6	88 (24)	20 (20)	68 (25)	0.391
RRT, *n* (%)	95 (11)	10 (4)	85 (14)	<0.001	37 (10)	7 (7)	30 (11)	0.342
Outcome								
LOS in hospital	9.8 (4.97, 17.68)	6.16 (2.98, 10.87)	11.71 (6.84, 20.22)	<0.001	9.6 (5.38, 15.69)	7.53 (3.1, 11.84)	10.46 (6, 17.36)	<0.001
LOS in ICU	2.64 (1.31, 5.92)	1.21 (0.88, 1.94)	3.66 (2.01, 8.54)	<0.001	2.87 (1.32, 6.71)	1.21 (0.94, 2.22)	4.04 (1.98, 8.12)	<0.001
28-death, *n* (%)	134 (16)	12 (5)	122 (20)	<0.001	57 (15)	7 (7)	50 (19)	0.010

Values are mean ± SD, *n* (%), or median (IQR). RBC, red blood cell; WBC, white blood cell; ALT, aspartate aminotransferase; AST, aspartate aminotransferase; CK-MB, Creatine kinase isoenzyme MB; ALP, Alkaline phosphatase; BUN, blood urea nitrogen; FBG, fasting blood glucose; TG, triglyceride; INR, International Normalized Ratio; PT, prothrombin time; PTT, partial prothrombin time. BMI, body mass index; SBP, systolic blood pressure; DBP, diastolic blood pressure; CKD, chronic kidney disease; COPD, chronic obstructive pulmonary disease; PCI, percutaneous coronary intervention; CABG, coronary artery bypass grafting; RRT, renal replacement therapy; LOS, length of stay.

Among the 9,042 patients initially identified with AMI, 2,927 were removed as duplicates, and 4,888 (54.1%) were excluded due to missing triglyceride or glucose values. To evaluate the impact of these exclusions, we compared key baseline variables between the included and excluded cohorts. Although some variables showed statistically significant differences (*p* < 0.05), all standardized mean differences (SMDs) were < 0.1, suggesting minimal practical imbalance. For example, the mean SOFA score differed by only 0.31 points on average, which is unlikely to affect clinical interpretation. These results suggest a low risk of selection bias, with details are presented in Supplementary Table S2.

### 3.2 Feature selection

**Figure 2:**
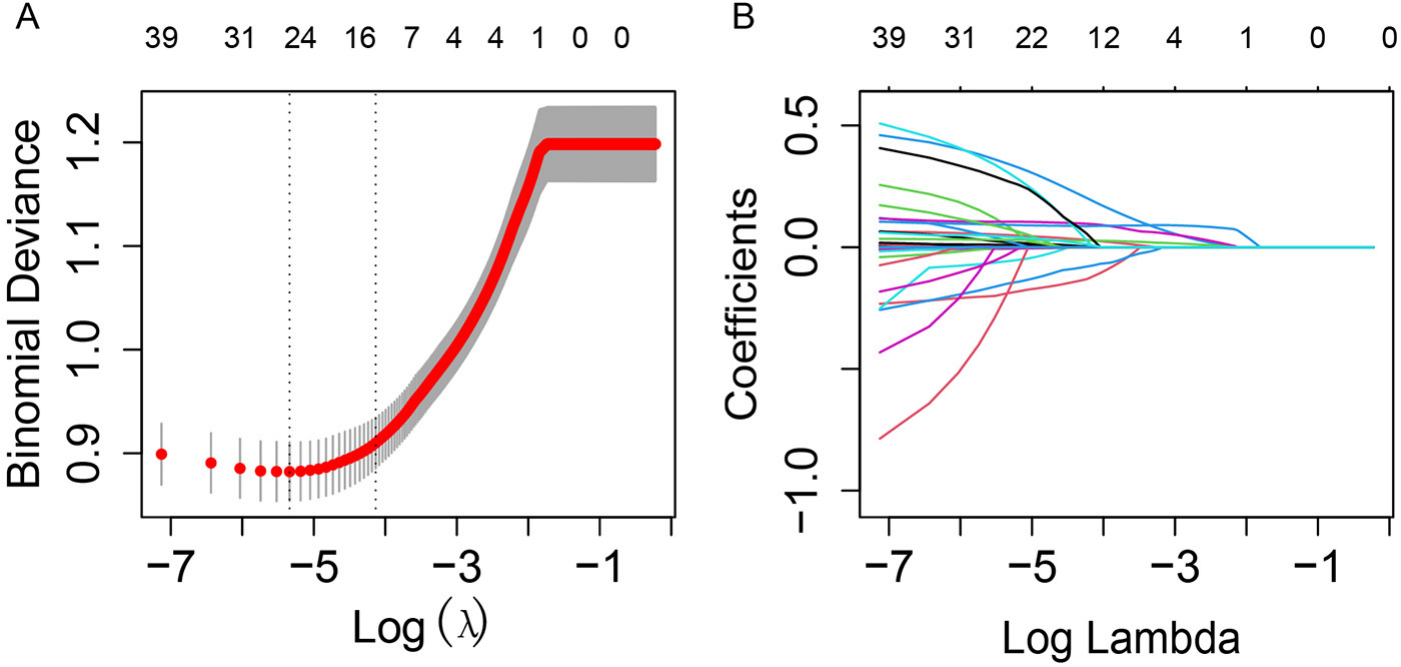
LASSO regression for variable selection with 5-fold cross-validation. **(A)** LASSO regression cross-validation curve. The optimal *λ* value was selected using 5-fold cross-validation in training cohort. **(B)** Path diagram of the LASSO coefficients. Each curve illustrates the trajectory of the coefficients for each variable as *λ* changes. The vertical axis represents the coefficient values, the lower horizontal axis shows log(*λ*), and the upper horizontal axis indicates the number of non-zero variables included in the model at each *λ* value.

Feature selection was performed using LASSO regression with 5-fold cross-validation to identify the most relevant predictors from the initial set of 43 variables. The optimal regularization parameter (lambda) was selected based on the minimum cross-validated error (lambda = 0.02). This process retained 17 variables with non-zero coefficients, including clinical scores (LODS, OASIS, SOFA), treatment procedures (CABG), vital signs (DBP, SBP, heart rate), laboratory parameters (ALP, AST, albumin, creatinine, glucose, sodium, BUN, hemoglobin), and demographic/clinical characteristics (age, BMI, LOS in ICU, CKD, hypertension, TyG index) (Figure 2). The corresponding coefficients for these predictors are presented in Supplementary Table S3.

Univariate logistic regression analysis of these variables revealed that 12 variables were significantly associated with AKI risk (*p* < 0.05). Subsequent multivariate logistic regression analysis identified 8 independent predictors of AKI, including TyG index, BUN, SOFA score, LOS in ICU, age, albumin, sodium, and SBP (Table 2). Among these, the TyG index demonstrated the strongest association with AKI risk, with each unit increase associated with a 31% higher risk of AKI (OR: 1.31, 95% CI: 1.07–1.60, *p* = 0.01). The full results of the multivariate analysis, including odds ratios (ORs) and 95% confidence intervals (CIs) for all predictors, are presented in Table 2.

**Table 2:** Univariate and multivariate regression analysis of screening variables.

Variables	Univariate analysis			Multivariate analysis		
	OR	95% CI	*P* value	OR	95% CI	*P* value
Age	1.01	1–1.02	0.037	1.01	1–1.03	0.036
Sodium	1.03	1–1.06	0.025	1.06	1–1.17	0.041
Creatinine	1.06	0.97–1.16	0.202			
Albumin	0.31	0.24–0.41	<0.001	0.82	0.78–0.98	0.042
BUN	1.01	1–1.02	0.005	1.21	1.08–1.32	<0.001
AST	1.01	1.01–1.03	<0.001	1.01	0.91–1.05	0.184
ALP	1.02	0.98–1.04	0.45			
Heart rate	1.01	1–1.02	0.01	0.97	0.95–1.01	0.109
SBP	0.98	0.97–0.99	<0.001	0.99	0.98–1.0	0.104
Hemoglobin	0.91	0.86–0.97	0.002	1.05	0.97–1.15	0.215
SOFA	1.33	1.27–1.4	<0.001	1.11	1.03–1.2	0.006
OASIS	1.11	1.09–1.13	<0.001	1.02	0.99–1.05	0.145
LODS	1.42	1.34–1.51	<0.001	1.15	1.04–1.27	0.008
CKD	1.47	1.1–1.97	0.01	1.41	0.97–2.06	0.074
CABG	1.16	0.87–1.55	0.312			
Hypertension	0.89	0.69–1.14	0.339			
TyG	1.49	1.25–1.76	<0.001	1.32	1.06–1.64	0.012
Glucose	1.01	1–1.02	0.044	0.91	0.86–1.07	0.195
LOS in ICU	1.67	1.52–1.84	<0.001	1.53	1.39–1.7	<0.001

OR, odds ratio; CI, confidence interval.

To evaluate the relationship between the TyG index and the risk of AKI, restricted cubic spline (RCS) analysis with four equally spaced knots was performed. The likelihood ratio test comparing the linear and spline models showed no evidence of nonlinearity (*P* for nonlinearity = 0.5094), indicating a linear association between TyG and AKI risk. Therefore, TyG was modeled as a continuous linear variable in the final prediction model (Supplementary Figure S1).

### 3.3 Model development and validation

**Figure 3:**
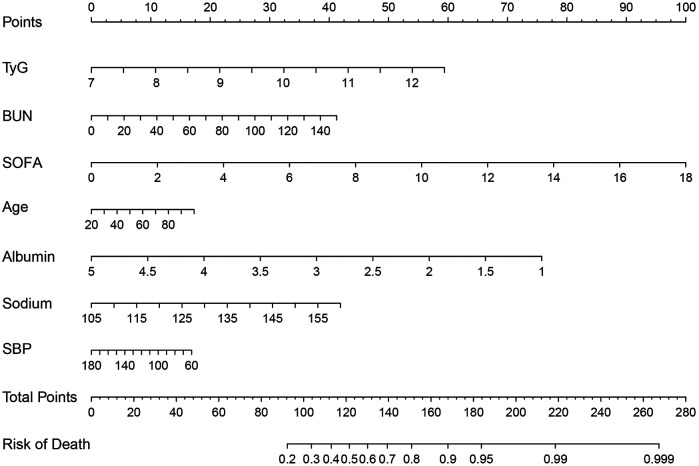
Nomogram for predicting the risk of AKI in patients with AMI.

Based on the results of multivariate logistic regression, a nomogram was constructed to estimate the risk of acute kidney injury (AKI) in ICU patients. The initial model incorporated eight independent predictors, including age, Sodium, albumin, LOS_ICU, heart rate, BUN, SBP, and the TyG index. To avoid potential reverse causality between LOS_ICU and the development of AKI, since AKI may itself prolong ICU stay, we excluded LOS_ICU from the final predictive model. To assess the potential impact of excluding LOS_ICU, we compared the predictive performance of the final model with and without this variable. The results showed no statistically significant difference in AUC across training, internal validation, and external validation cohorts. Detailed comparison results are provided in Supplementary Table S4. Each variable was assigned a point value proportional to its contribution, and the total score was mapped to the corresponding probability of AKI. This graphical tool provides an intuitive and practical means for individualized risk assessment and clinical decision-making (Figure 3). To further facilitate clinical use, we plan to convert the content of Figure 3 into a list of point values and include it in the Supplementary Table S5.

**Figure 4:**
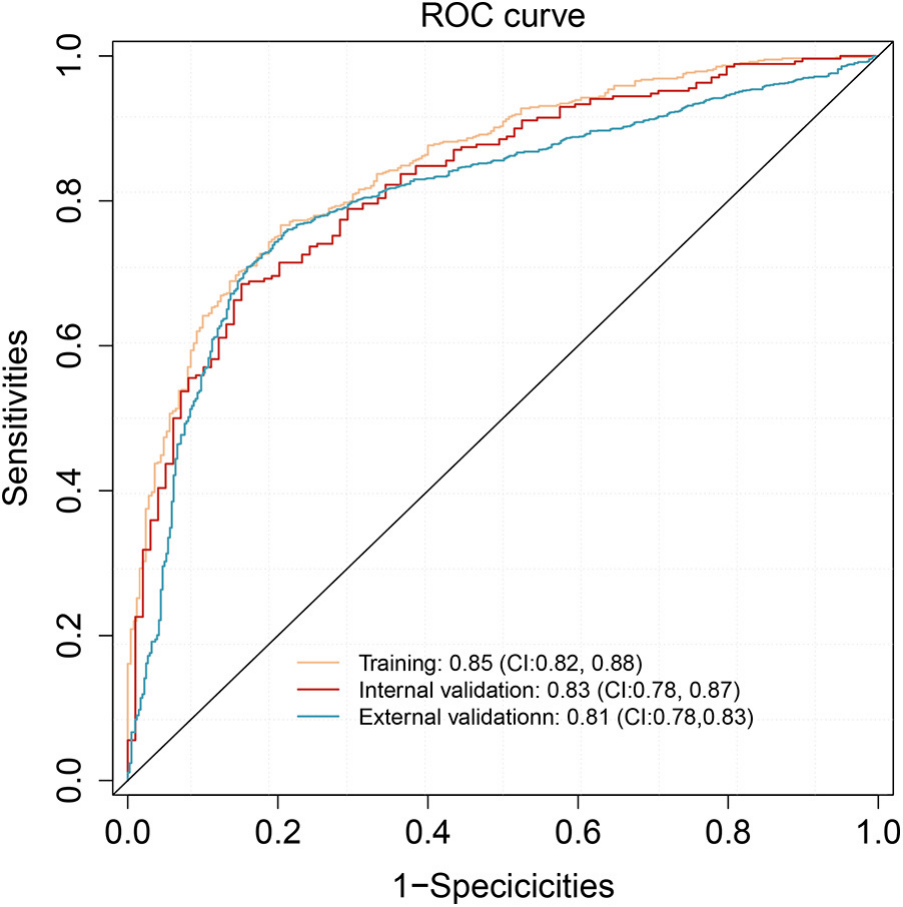
ROC analysis of the nomogram model in training, internal validation, and external validation cohorts.

The predictive performance of the TyG model was evaluated using the area under the receiver operating characteristic curve (AUC). In the training cohort, the model exhibited excellent discrimination, with an AUC of 0.85 (95% CI: 0.82–0.88). This performance was maintained in the internal validation cohort, with an AUC of 0.83 (95% CI: 0.78–0.87), and further confirmed in the external validation cohort, achieving an AUC of 0.81 (95% CI: 0.78–0.83), indicating good generalizability (Figure 4). The detailed classification performance of the TyG model across different cohorts is presented in Table 3. In the training cohort, the model demonstrated robust predictive capability, achieving a recall of 0.88, precision of 0.83, and an F1 score of 0.85, with an overall accuracy of 0.79. These performance metrics remained consistent in the internal validation cohort (recall = 0.87, F1 score = 0.86, precision = 0.84) and the external validation cohort (recall = 0.84, F1 score = 0.86, precision = 0.84), indicating stable performance across different populations.

**Table 3:** Performance comparison of TyG and non-TyG models across training, internal validation and external validation cohorts.

Model	Cohorts	AUC	Recall	F1 score	Accuracy	Precision	*P* value
TyG	Training	0.85	0.88	0.85	0.79	0.83	<0.001
	Internal validation	0.83	0.87	0.86	0.79	0.84	<0.001
	External validation	0.81	0.84	0.86	0.78	0.84	0.032
non-TyG	Training	0.77	0.89	0.83	0.75	0.78	-
	Internal validation	0.76	0.86	0.86	0.77	0.78	-
	External validation	0.76	0.85	0.83	0.76	0.80	-

**Figure 5:**
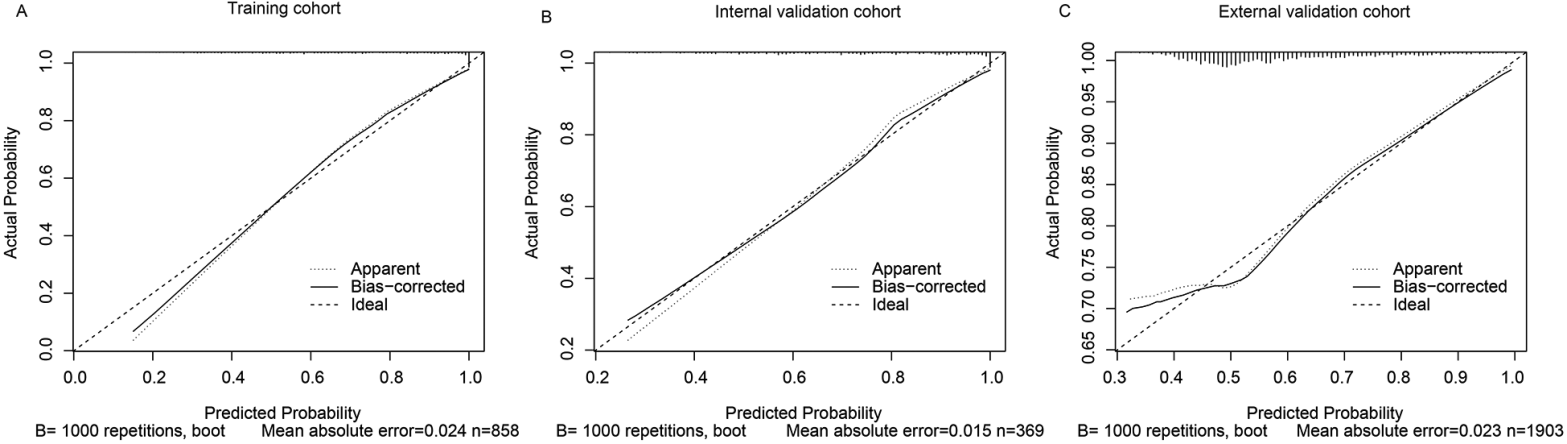
Calibration curves of the predictive model for AKI in AMI patients. **(A)** Training cohort, **(B)** Internal validation cohort, **(C)** External validation cohort. The closer the calibration curve is to the diagonal line, the better the agreement between predicted and observed outcomes.

The calibration curve was used to assess the agreement between predicted probabilities and observed outcomes. In the training set (*n* = 858), the mean absolute error (MAE) was 0.024 with a Hosmer-Lemeshow *χ*^2^ = 5.07 (*p* = 0.749), indicating no significant deviation from perfect calibration. This was confirmed in the validation cohorts (internal: MAE = 0.015, *χ*^2^ = 2.74, *p* = 0.783; external: MAE = 0.023, *χ*^2^ = 4.42, *p* = 0.357) (Figure 5).

Twelve patients were identified who died before the onset of AKI. After excluding these patients, the model was re-applied, and AUC values showed no significant change (Training cohort: 0.854 vs. Adjusted cohort: 0.849, *p* = 0.7921). This indicates the model's discrimination was not sensitive to early mortality bias (shown in Supplementary Table S6).

### 3.4 Comparative analysis of TyG and non-TyG models

The incremental predictive value of the TyG model was assessed using both the net reclassification index (NRI) and integrated discrimination improvement (IDI). In the training cohort, the model demonstrated substantial improvements, with an NRI of 0.224 (95% CI: 0.160–0.287) and IDI of 0.112 (95% CI: 0.094–0.129). These improvements remained statistically significant in the internal validation cohort (NRI = 0.183, 95% CI: 0.083–0.282; IDI = 0.087, 95% CI: 0.062–0.112). The TyG model demonstrated statistically significant incremental predictive value compared to the non-TyG model in the external validation cohort (NRI = 0.033, 95% CI: 0.013–0.054; IDI = 0.006, 95% CI: 0.003–0.008), with smaller effect sizes than observed in the training cohort. The complete results are presented in Table 4.

**Table 4:** Comparison of the non-TyG and TyG models through NRI and IDI.

Cohorts	NRI	95% CI	*P* value	IDI	95% CI	*P* value
Trainning validation	0.224	0.160, 0.287	<0.01	0.112	0.094, 0.129	<0.01
Internal validation	0.183	0.083, 0.282	<0.01	0.087	0.064, 0.112	<0.01
External validation	0.033	0.013, 0.054	0.001	0.006	0.003, 0.008	<0.01

**Figure 6:**
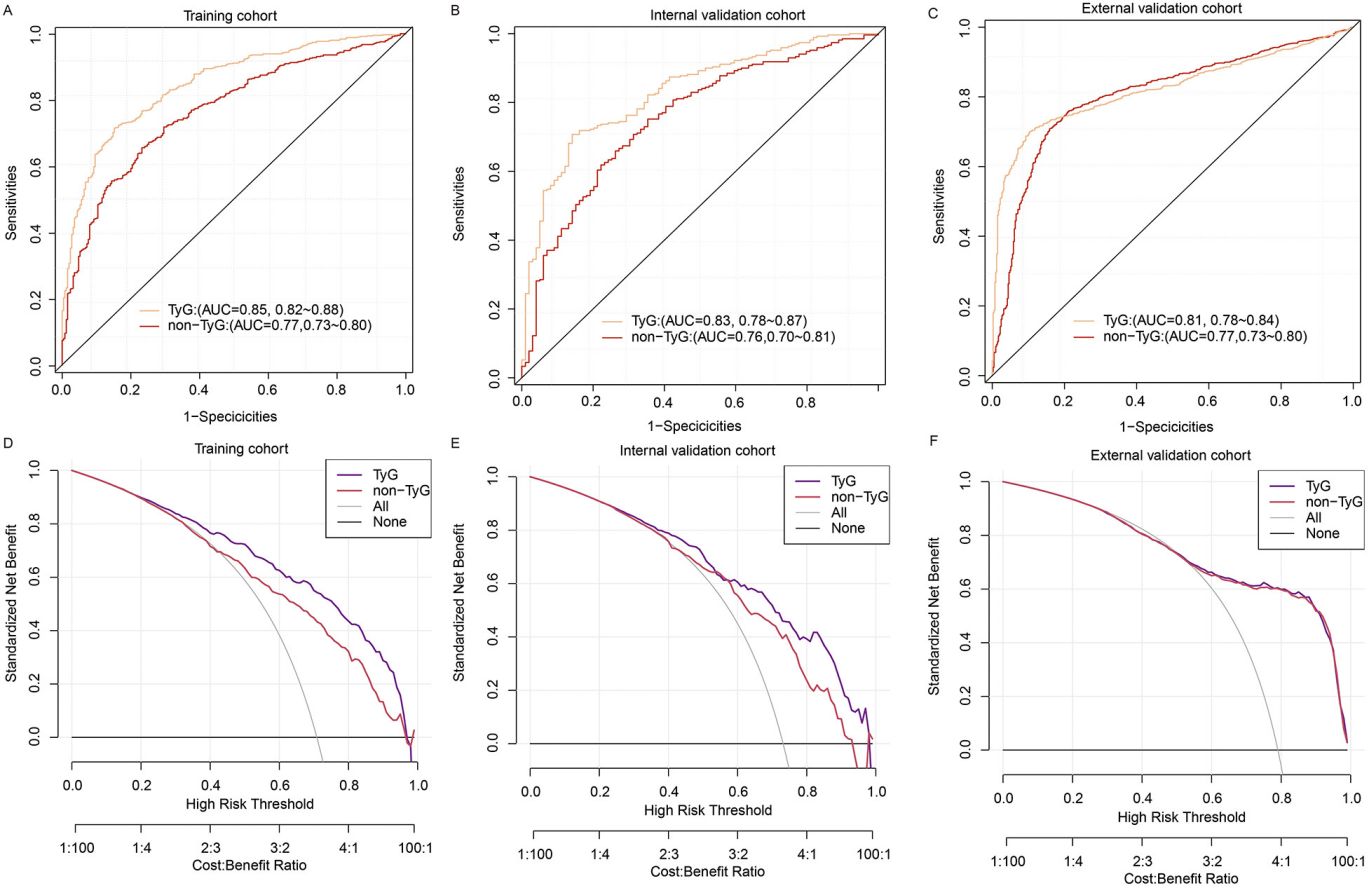
Comparison of TyG and non-TyG models using ROC and DCA across datasets. **(A–C)** ROC curve comparisons between the TyG model and the non-TyG model in the training cohort **(A)**, internal validation cohort **(B)**, and external validation cohort **(C–F)** DCA comparing the net clinical benefits of the TyG model and non-TyG model across varying threshold probabilities in the training **(D)**, internal validation **(E)**, and external validation **(F)** cohorts.

The TyG model demonstrated statistically significant incremental predictive value compared to the non-TyG model across all evaluation cohorts. In the training cohort, the AUC improved from 0.77 (95% CI: 0.73–0.80) to 0.85 (0.82–0.88; *p* < 0.001), with corresponding enhancements in recall (0.90 vs. 0.85) and precision (0.84 vs. 0.78). This performance advantage persisted in internal validation (AUC 0.83 vs. 0.76, *p* < 0.001) and remained statistically significant in external validation (AUC 0.81 vs. 0.76, *p* = 0.032), though with attenuated effect sizes (Figures 6A–C; Table 3).

Decision curve analysis revealed differential net benefit profiles between the models across cohorts (Figures 6D–F). In the training cohort, the TyG model demonstrated superior net benefit at lower risk thresholds (22%–45% probability range), while both models achieved comparable utility above 45% risk probability. This pattern persisted in internal validation, with the TyG model maintaining an advantage in the 25%–45% threshold range. Notably, in external validation, the net benefit curves of both models converged completely, showing identical clinical utility across all decision thresholds (55%–100%).

## 4 Discussion

AKI is one of the most frequent complications of AMI, often occurring early during hospitalization and significantly worsening both short- and long-term outcomes (20). However, current diagnostic criteria rely on delayed changes in serum creatinine or urine output, limiting timely clinical intervention. Therefore, accurate and early clinical prediction models are urgently needed to guide risk stratification in AMI patients.

In this study, we developed and validated a prognostic nomogram to predict AKI risk in patients with AMI. To our knowledge, this is the first study to incorporate the TyG index into an AKI prediction model specifically designed for AMI patients. This novel integration highlights the potential clinical utility of TyG as an early metabolic marker for renal risk stratification in the setting of acute cardiovascular events. As a novel and practical surrogate marker of insulin resistance (IR), the TyG index may capture early metabolic disturbances that precede overt renal dysfunction.

IR is increasingly recognized as a central pathophysiological factor linking cardiometabolic disorders with renal impairment (8, 9). It contributes to atherogenic dyslipidemia, characterized by elevated triglycerides, reduced high-density lipoprotein (HDL), and increased small dense low-density lipoprotein (LDL) particles. These lipid abnormalities promote oxidative stress and endothelial dysfunction, impairing vasodilatory capacity and facilitating vascular inflammation (21). Endothelial dysfunction subsequently disrupts the regulation of renal perfusion and filtration, increasing the susceptibility to AKI during hemodynamic insults such as AMI.

Additionally, IR-induced hyperglycemia upregulates the expression of angiotensinogen, angiotensin-converting enzyme (ACE), and angiotensin II, leading to overactivation of the renin–angiotensin–aldosterone system (RAAS). This activation promotes systemic vasoconstriction, sodium retention, and intraglomerular hypertension, thereby increasing renal hemodynamic stress and accelerating nephron injury (22). In parallel, concurrent hyperinsulinemia further stimulates the mitogen-activated protein kinase (MAPK) signaling pathway, which exacerbates local inflammation, vascular remodeling, and tubular injury in renal tissues (23). These converging processes create a pro-inflammatory and pro-fibrotic milieu that underlies both acute and chronic kidney injury.

Together, these mechanisms provide a robust pathophysiological foundation for the role of IR in endothelial injury, organ dysfunction, and the development of AKI, particularly in patients with AMI. Although the homeostasis model assessment of insulin resistance (HOMA-IR) is widely used to estimate insulin resistance in clinical and research settings, its application is limited by the need for fasting insulin measurements and relatively complex procedures (24). In contrast, the TyG index provides a simpler and more reproducible surrogate, with multiple studies demonstrating comparable predictive performance to HOMA-IR in assessing metabolic and cardiovascular risks (25–27). These advantages make the TyG index a practical alternative for routine clinical use.

In this study, we implemented LASSO-based predictor selection to identify robust clinical variables from high-dimensional data, followed by univariate and multivariate validation. This process selected the TyG index and seven additional clinically interpretable predictors for the final model. Our findings align with numerous multicenter studies demonstrating the TyG index's predictive value for AKI across clinical scenarios. Recent analyses validate elevated AKI risks with higher TyG levels in diabetic and hypertensive critically ill populations (28, 29). Similarly, Zhang et al. (30). demonstrated that higher TyG index significantly increased AKI risk in 1,501 coronary artery disease patients (HR 1.62, 95% CI 1.15–2.27), with even stronger associations in non-diabetic subgroups. In addition, a large cohort study of 1,426 septic patients further supported the prognostic value of TyG, showing significant associations with both sepsis-associated AKI risk (OR 1.40, 95% CI 1.14–1.73) and extended length of hospital stay (*β* = 1.79 days) (29). Given the consistent evidence linking elevated TyG levels with AKI risk in diverse populations, including patients with diabetes, coronary artery disease, and sepsis, the inclusion of the TyG index in our predictive model is both biologically plausible and clinically justified. In our study cohort of AMI patients, the TyG index remained an independent predictor of AKI after adjustment for confounding factors, suggesting that its predictive value extends beyond its role as a marker of metabolic dysfunction. These results provide a strong rationale for selecting the TyG index as a key variable in predictive model construction.

In addition to the TyG index, our model incorporates seven other variables that have demonstrated independent associations with AKI in prior studies. These predictors were selected not only for their statistical significance, but also for their established links to renal pathophysiology. Each variable reflects a distinct clinical domain relevant to AKI risk and has been previously associated with adverse renal outcomes in critically ill or cardiovascular populations. Elevated blood urea nitrogen (BUN) is a well marker of renal dysfunction, reflecting impaired nitrogenous waste excretion and reduced glomerular filtration rate (31). In patients with myocardial infarction, sustained renal hypoperfusion due to low cardiac output or hypotension leads to acute kidney injury (32), where BUN elevation may better reflect the severity of renal dysfunction compared to serum creatinine or eGFR measurements (33). Low albumin has been consistently identified as an independent risk factor for AKI, as it contributes to reduced oncotic pressure, increased vascular permeability, and systemic inflammation, thereby promoting renal hypoperfusion and tubular injury (13, 34). The Sequential Organ Failure Assessment (SOFA) score reflects the overall severity of organ dysfunction. Its association with AKI has been widely reported, with higher scores indicating an increased systemic burden and a higher risk of renal impairment in AMI (13, 35). Length of ICU stay (LOS_ICU) reflects illness severity and is associated with prolonged exposure to nephrotoxic medications, hemodynamic instability, and cumulative intervention burden, all of which are established risk factors for AKI development (5). Serum sodium levels, particularly in the context of hyponatremia or dysnatremia, have been associated with poor renal outcomes. These disturbances may reflect underlying fluid imbalance, neurohormonal dysregulation, or renal salt-wasting, all of which are relevant to AKI pathogenesis (36). Our model consistently identified age as an independent predictor of AKI, aligning with established clinical evidence of increased risk in older populations (37). This association likely reflects progressive age-related declines in renal functional reserve and vascular compliance. Consistent with our findings, several studies have established the relationship between SBP and AKI occurrence across various clinical contexts (32, 38). This underscores the importance of maintaining optimal perfusion pressure in critically ill patients. Transient hypotensive episodes may compromise renal perfusion, initiating a cascade of events that ultimately lead to AKI development (39). By integrating these clinically relevant and biologically plausible variables, our model adopts a multidimensional perspective that encompasses metabolic, immune status, hemodynamic, and organ function domains. This comprehensive approach enhances its clinical utility for early identification of patients at risk for AKI following AMI, allowing timely preventive strategies and targeted interventions.

Our model incorporating the TyG index demonstrated strong discriminative performance, with AUC values of 0.85 in the training cohort, 0.83 in the internal validation cohort, and 0.80 in the external validation cohort. The slight decrease in AUC in the external cohort (0.80 vs. 0.83 in internal validation) may reflect inherent heterogeneity between populations, such as differences in baseline characteristics, regional treatment protocols, or data collection methods. Future studies should further explore these factors to enhance model generalizability. Several existing models have attempted to predict AKI following AMI, but most are limited by either lack of external validation or reliance on late-occurring variables. For instance, the study by Xun W et al. (40) developed a model incorporating eGFR, hemoglobin, sodium, bicarbonate, total bilirubin, age, diabetes, and heart failure, achieving a relatively high AUC of 0.86. However, the model was developed and validated in a single-center cohort without external validation, raising concerns about its generalizability. Similarly, the widely used Mehran score (41) was specifically designed for PCI populations and includes post-procedural variables such as contrast volume and hypotension, limiting its applicability in AMI patients who do not undergo PCI or require early risk stratification. In contrast, the model proposed by Bo X et al. (42), with an AUC of 0.76, included factors such as in-hospital shock and maximum furosemide dosage, which occur later during hospitalization and are thus less suitable for early AKI prediction.

To evaluate the incremental predictive performance of the TyG index, we compared the TyG and non-TyG models across the training, internal validation, and external validation cohorts. The TyG model consistently demonstrated superior discriminative ability, with significantly higher AUC values across all cohorts (*p* < 0.05). To further quantify the improvement in risk prediction, we performed Net Reclassification Improvement (NRI) and Integrated Discrimination Improvement (IDI) analyses. The TyG model yielded significantly positive NRI and IDI values in all cohorts, particularly in the training (NRI: 0.2238; IDI: 0.1115) and internal validation (NRI: 0.1421; IDI: 0.0949) cohorts, indicating substantial improvements in risk classification and discrimination. Although the NRI and IDI remained statistically significant in the external validation cohort, the magnitude of improvement (NRI: 0.0302; IDI: 0.0058) was modest. In accordance with these results, decision curve analysis (DCA) demonstrated greater clinical net benefit for the TyG-based model in the training and internal validation sets, whereas the benefit was less pronounced in the external cohort. These results underscore the added value of the TyG index in early AKI risk stratification, but also highlight the need for further external validation in diverse populations and prospective settings to confirm its generalizability and real-world clinical utility.

## 5 Study limitation

This study has several limitations. First, due to its retrospective design, residual confounding cannot be entirely ruled out, despite adjustment for multiple clinically relevant variables. Some important factors such as nephrotoxic drug exposure, fluid balance, and medication use were not consistently available in the databases. Second, the TyG index was calculated from a single fasting measurement upon ICU admission, which may not fully reflect dynamic metabolic changes during hospitalization. Third, the use of enteral or parenteral nutrition during hospitalization may influence lipid and glucose metabolism, potentially resulting in an elevated TyG index. Although this effect cannot be entirely excluded, the large sample size in our study likely attenuates its overall impact. Further investigations are needed to elucidate the mechanisms linking insulin resistance to acute kidney injury in patients with acute myocardial infarction. Fourth, CK-MB, a clinically important marker of myocardial infarction severity, had a missing rate of 33.3%. We categorized CK-MB into clinically meaningful intervals and treated missing values as a separate category rather than performing multiple imputation. While this approach preserved the variable's predictive value without unverifiable assumptions about the missing data mechanism, it may still introduce residual bias. Future studies with more complete data are warranted to address this issue. Fifth, the AKI assessments in the MIMIC-IV database begin at ICU admission, and no pre-ICU AKI status was recorded. Thus, we could not determine whether some patients had already developed AKI before ICU entry, which may have introduced misclassification bias. Additionally, the possibility of pseudo-worsening of renal function in AKI patients cannot be excluded. This is often related to the use of nephrotoxic drugs or negative fluid balance, which were not fully captured in our dataset. These factors may have contributed to AKI misclassification and residual confounding, and should be taken into consideration when interpreting our findings. Finally, while the model showed good discrimination and calibration across all cohorts, its real-world clinical utility remains to be validated in prospective settings.

## 6 Conclusion

Our study developed and externally validated a novel nomogram incorporating the TyG index for predicting AKI in patients with AMI. The model demonstrated robust discriminative ability, strong clinical utility, and excellent generalizability across multiple cohorts. The integration of the TyG index as a surrogate of insulin resistance highlights its potential role in early AKI risk stratification and personalized patient management.

## Data Availability

The raw data supporting the conclusions of this article will be made available by the authors, without undue reservation.

## References

[1] Benjamin EJ Virani SS Callaway CW Chamberlain AM Chang AR Cheng S Heart disease and stroke statistics-2018 update: a report from the American Heart Association. Circulation. (2018) 137(12):e67–e492. 10.1161/cir.0000000000000558 29386200

[2] Miura T Kuno A Tanaka M. Diabetes modulation of the myocardial infarction-acute kidney injur*y* axis. Am J Physiol Heart Circ Physiol. (2022) 322(3):H394–h405. 10.1152/ajpheart.00639.2021 35089809

[3] Marenzi G Cabiati A Bertoli SV Assanelli E Marana I De Metrio M Incidence and relevance of acute kidney injury in patients hospitalized with acute coronary syndromes. Am J Cardiol. (2013) 111(6):816–22. 10.1016/j.amjcard.2012.11.046 23273525

[4] Mezhonov EM Vialkina IA Vakulchik KA Shalaev SV. Acute kidney injury in patients with ST-segment elevation acute myocardial infarction: predictors and outcomes. Saudi J Kidney Dis Transpl. (2021) 32(2):318–27. 10.4103/1319-2442.335442 35017324

[5] Maksimczuk J Galas A Krzesinski P. What promotes acute kidney injury in patients with myocardial infarction and multivessel coronary artery disease-contrast Media, hydration Status or something else? Nutrients. (2022) 15(1):21. 10.3390/nu15010021 36615678 PMC9824824

[6] Lee SH Park SY Choi CS. Insulin resistance: from mechanisms to therapeutic strategies. Diabetes Metab J. (2022) 46(1):15–37. 10.4093/dmj.2021.0280 34965646 PMC8831809

[7] Chen CL Liu L Lo K Huang JY Yu YL Huang YQ Association between triglyceride glucose index and risk of new-onset diabetes among Chinese adults: findings from the China health and retirement longitudinal study. Front Cardiovasc Med. (2020) 7:610322. 10.3389/fcvm.2020.610322 33330672 PMC7728664

[8] Demir S Nawroth PP Herzig S Ekim Ustunel B. Emerging targets in type 2 diabetes and diabetic complications. Adv Sci (Weinh). (2021) 8(18):e2100275. 10.1002/advs.202100275 34319011 PMC8456215

[9] Artunc F Schleicher E Weigert C Fritsche A Stefan N Haring HU. The impact of insulin resistance on the kidney and vasculature. Nat Rev Nephrol. (2016) 12(12):721–37. 10.1038/nrneph.2016.145 27748389

[10] Cai D Xiao T Chen Q Gu Q Wang Y Ji Y Association between triglyceride glucose and acute kidney injury in patients with acute myocardial infarction: a propensity score-matched analysis. BMC Cardiovasc Disord. (2024) 24(1):216. 10.1186/s12872-024-03864-5 38643093 PMC11031878

[11] Shi Y Duan H Liu J Shi X Zhao M Zhang Y. Association of triglyceride glucose index with the risk of acute kidney injury in patients with coronary revascularization: a cohort study. Diabetol Metab Syndr. (2024) 16(1):117. 10.1186/s13098-024-01358-0 38807249 PMC11131318

[12] Avagimyan A Pogosova N Fogacci F Aghajanova E Djndoyan Z Patoulias D Triglyceride-glucose index (TyG) as a novel biomarker in the era of cardiometabolic medicine. Int J Cardiol. (2025) 418:132663. 10.1016/j.ijcard.2024.132663 39426418

[13] Tao F Yang H Wang W Bi X Dai Y Zhu A Acute kidney injury prediction model utility in premature myocardial infarction. iScience. (2024) 27(3):109153. 10.1016/j.isci.2024.109153 38390493 PMC10882170

[14] Shi Y Yu C. U shape association between triglyceride glucose index and congestive heart failure in patients with diabetes and prediabetes. Nutr Metab (Lond). (2024) 21(1):42. 10.1186/s12986-024-00819-7 38956581 PMC11221084

[15] Johnson A Bulgarelli L Pollard T Gow B Moody B Horng S “MIMIC-IV” (version 3.1). *PhysioNet*. (2024).

[16] Pollard TJ Johnson AEW Raffa JD Celi LA Mark RG Badawi O. The eICU collaborative research database, a freely available multi-center database for critical care research. Sci Data. (2018) 5:180178. 10.1038/sdata.2018.178 30204154 PMC6132188

[17] Johnson AEW Bulgarelli L Shen L Gayles A Shammout A Horng S Author correction: MIMIC-IV, a freely accessible electronic health record dataset. Sci Data. (2023) 10(1):219. 10.1038/s41597-023-02136-9 37072428 PMC10113185

[18] Yang Z Gong H Kan F Ji N. Association between the triglyceride glucose (TyG) index and the risk of acute kidney injury in critically ill patients with heart failure: analysis of the MIMIC-IV database. Cardiovasc Diabetol. (2023) 22(1):232. 10.1186/s12933-023-01971-9 37653418 PMC10472684

[19] Khwaja A. KDIGO Clinical practice guidelines for acute kidney injury. Nephron Clin Pract. (2012) 120(4):c179–84. 10.1159/000339789 22890468

[20] El-Ahmadi A Abassi MS Andersson HB Engstrøm T Clemmensen P Helqvist S Acute kidney injury - a frequent and serious complication after primary percutaneous coronary intervention in patients with ST-segment elevation myocardial infarction. PLoS One. (2019) 14(12):e0226625. 10.1371/journal.pone.0226625 31860670 PMC6924683

[21] Ormazabal V Nair S Elfeky O Aguayo C Salomon C Zuñiga FA. Association between insulin resistance and the development of cardiovascular disease. Cardiovasc Diabetol. (2018) 17(1):122. 10.1186/s12933-018-0762-4 30170598 PMC6119242

[22] Zhou MS Schulman IH Zeng Q. Link between the renin-angiotensin system and insulin resistance: implications for cardiovascular disease. Vasc Med. (2012) 17(5):330–41. 10.1177/1358863X12450094 22814999

[23] Zhou MS Schulman IH Raij L. Vascular inflammation, insulin resistance, and endothelial dysfunction in salt-sensitive hypertension: role of nuclear factor kappa B activation. J Hypertens. (2010) 28(3):527–35. 10.1097/HJH.0b013e3283340da8 19898250

[24] Park HM Lee HS Lee YJ Lee JH. The triglyceride-glucose index is a more powerful surrogate marker for predicting the prevalence and incidence of type 2 diabetes mellitus than the homeostatic model assessment of insulin resistance. Diabetes Res Clin Pract. (2021) 180:109042. 10.1016/j.diabres.2021.109042 34506839

[25] Guerrero-Romero F Simental-Mendia LE Gonzalez-Ortiz M Martinez-Abundis E Ramos-Zavala MG Hernandez-Gonzalez SO The product of triglycerides and glucose, a simple measure of insulin sensitivity. Comparison with the euglycemic-hyperinsulinemic clamp. J Clin Endocrinol Metab. (2010) 95(7):3347–51. 10.1210/jc.2010-0288 20484475

[26] Vasques AC Novaes FS de Oliveira Mda S Souza JR Yamanaka A Pareja JC Tyg index performs better than HOMA in a Brazilian population: a hyperglycemic clamp validated study. Diabetes Res Clin Pract. (2011) 93(3):e98–e100. 10.1016/j.diabres.2011.05.030 21665314

[27] Unger G Benozzi SF Perruzza F Pennacchiotti GL. Triglycerides and glucose index: a useful indicator of insulin resistance. Endocrinol Nutr. (2014) 61(10):533–40. 10.1016/j.endonu.2014.06.009 25174769

[28] Zhang W Yang Z. Association between the triglyceride glucose index and the risk of acute kidney injury in critically ill patients with hypertension: analysis of the MIMIC-IV database. Front Endocrinol (Lausanne). (2024) 15:1437709. 10.3389/fendo.2024.1437709 39072271 PMC11272463

[29] Jin Z Zhang K. Association between triglyceride-glucose index and AKI in ICU patients based on MIMICIV database: a cross-sectional study. Ren Fail. (2023) 45(1):2238830. 10.1080/0886022x.2023.2238830 37563796 PMC10424620

[30] Zhang Y Li G Li J Jian B Wang K Chen J The triglyceride-glucose index and acute kidney injury risk in critically ill patients with coronary artery disease. Ren Fail. (2025) 47(1):2466818. 10.1080/0886022x.2025.2466818 39972619 PMC11843639

[31] Nong Y Wei X Qiu H Yang H Yang J Lu J Analysis of risk factors for severe acute kidney injury in patients with acute myocardial infarction: a retrospective study. Front Nephrol. (2023) 3:1047249. 10.3389/fneph.2023.1047249 37675384 PMC10479598

[32] Song MJ Lee SH Leem AY Kim SY Chung KS Kim EY Predictors and outcomes of sepsis-induced cardiomyopathy in critically ill patients. Acute Crit Care. (2020) 35(2):67–76. 10.4266/acc.2020.00024 32407613 PMC7280797

[33] Khoury J Bahouth F Stabholz Y Elias A Mashiach T Aronson D Blood urea nitrogen variation upon admission and at discharge in patients with heart failure. ESC Heart Fail. (2019) 6(4):809–16. 10.1002/ehf2.12471 31199082 PMC6676277

[34] Hansrivijit P Yarlagadda K Cheungpasitporn W Thongprayoon C Ghahramani N. Hypoalbuminemia is associated with increased risk of acute kidney injury in hospitalized patients: a meta-analysis. J Crit Care. (2021) 61:96–102. 10.1016/j.jcrc.2020.10.013 33157311

[35] Folkestad T Brurberg KG Nordhuus KM Tveiten CK Guttormsen AB Os I Acute kidney injury in burn patients admitted to the intensive care unit: a systematic review and meta-analysis. Crit Care. (2020) 24(1):2. 10.1186/s13054-019-2710-4 31898523 PMC6941386

[36] Hoorn EJ Zietse R. Diagnosis and treatment of hyponatremia: compilation of the guidelines. J Am Soc Nephrol. (2017) 28(5):1340–9. 10.1681/asn.2016101139 28174217 PMC5407738

[37] Privratsky JR Fuller M Raghunathan K Ohnuma T Bartz RR Schroeder R Postoperative acute kidney injury by age and sex: a retrospective cohort association study. Anesthesiology. (2023) 138(2):184–94. 10.1097/aln.0000000000004436 36512724 PMC10439699

[38] Holder AL Gupta N Lulaj E Furgiuele M Hidalgo I Jones MP Predictors of early progression to severe sepsis or shock among emergency department patients with nonsevere sepsis. Int J Emerg Med. (2016) 9(1):10. 10.1186/s12245-016-0106-7 26908009 PMC4764600

[39] Shin IS Kim DK An S Gong SC Kim MH Rahman MH Biomarkers to predict multiorgan distress syndrome and acute kidney injury in critically ill surgical patients. Medicina (Kaunas, Lithuania). (2023) 59(12):2054. 10.3390/medicina59122054 38138157 PMC10744752

[40] Wang X Fu X. Predicting AKI in patients with AMI: development and assessment of a new predictive nomogram. Medicine (Baltimore). (2023) 102(24):e33991. 10.1097/md.0000000000033991 37327276 PMC10270522

[41] Sgura FA Bertelli L Monopoli D Leuzzi C Guerri E Spartà I Mehran contrast-induced nephropathy risk score predicts short- and long-term clinical outcomes in patients with ST-elevation-myocardial infarction. Circ Cardiovasc Interv. (2010) 3(5):491–8. 10.1161/circinterventions.110.955310 20923986

[42] Xu FB Cheng H Yue T Ye N Zhang HJ Chen YP. Derivation and validation of a prediction score for acute kidney injury secondary to acute myocardial infarction in Chinese patients. BMC Nephrol. (2019) 20(1):195. 10.1186/s12882-019-1379-x 31146701 PMC6543657

