# Supplementary material for: Development and validation of a triglyceride-glucose integrated nomogram for acute kidney injury prediction in acute myocardial infarction patients: a multicenter database study
Source: Front Cardiovasc Med. 2025 Sep 2;12:1620664. doi: 10.3389/fcvm.2025.1620664 (PMC12436489; doi:10.3389/fcvm.2025.1620664)
Supplement: Supplementary file 1 [file Datasheet1.docx]

Supplementary Material

# Supplementary Figures and Tables

## Supplementary Figures


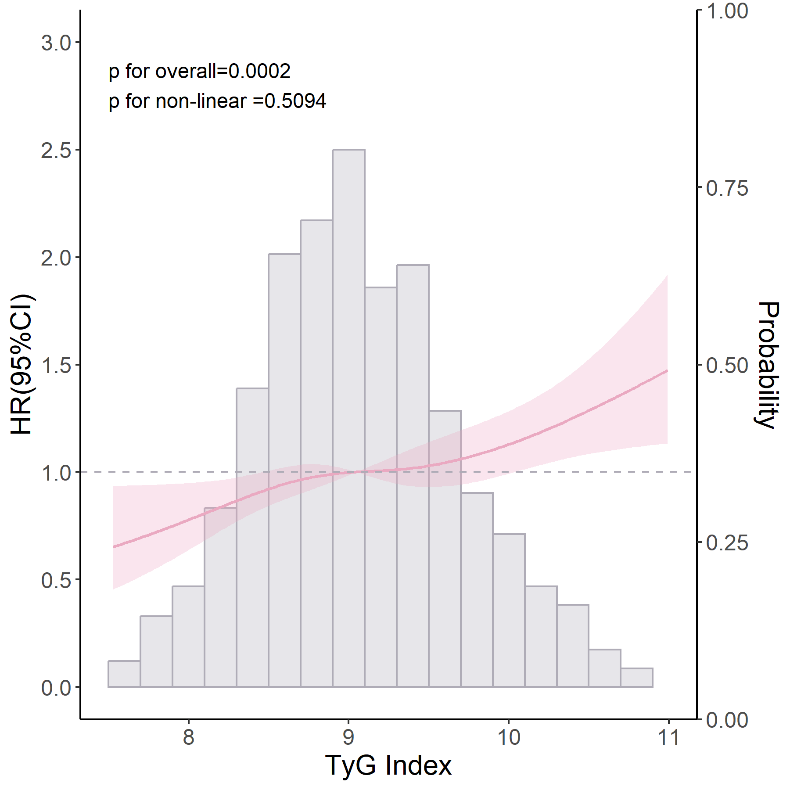


**Supplementary Figure 1.** RCS for TyG index and risk of acute kidney injury in patients with acute myocardial infarction. RCS: restricted cubic spline; HR: hazard ratio; CI: confidence interval; TyG: triglyceride–glucose.

## Supplementary Tables

**Supplementary Table 1.** Baseline characteristics of the external validation cohort.

| Supplementary Table 1. Baseline characteristics of the external validation cohort | | | | |
| --- | --- | --- | --- | --- |
| Variables | Total  (n = 1954) | Non-AKI  (n = 409) | AKI  (n = 1545) | *p* value |
| Albumin, g/dL | 3.52 ± 1.11 | 3.77 ± 1.28 | 3.45 ± 1.05 | < 0.001 |
| Age, years | 64.9 ± 13.21 | 63.62 ± 13.35 | 65.23 ± 13.16 | 0.03 |
| BUN, mg/dL | 18 (14, 25) | 17 (13, 21.2) | 19 (14, 27) | < 0.001 |
| LOS_ICU, days | 1.97 (1.41, 3.42) | 1.79 (1.3, 2.6) | 2.07 (1.46, 3.7) | < 0.001 |
| SOFA | 7.08 ± 4.53 | 2.86 ± 2.38 | 8.19 ± 4.3 | < 0.001 |
| TyG, (mg/dL)^2^ | 9.01 ± 0.79 | 8.19 ± 0.77 | 9.15 ± 0.77 | 0.058 |
| SBP, mmHg | 75.74 ± 35.25 | 88.51 ± 35.6 | 72.36 ± 34.38 | < 0.001 |
| Sodium, mEq/L | 138 (135, 140) | 138 (135, 140) | 138 (135, 140) | 0.71 |

**Supplementary Table 2. Comparison of baseline characteristics before and after exclusion of patients with missing triglyceride or glucose data.**

| Supplementary Table 2. Comparison of baseline characteristics before and after exclusion of patients with missing triglyceride or glucose data. | | | | |
| --- | --- | --- | --- | --- |
| variables | Full cohort (before exclusion) | Final cohort (after exclusion) | p value | SMD |
| Age, (years) mean±sd | 67.98±11.78 | 67.2±12.61 | 0.047 | 0.064 |
| Gender |  |  | 0.057 | 0.061 |
| Female, n (%) | 2048(33.49%) | 376 (30.64%) |  |  |
| Male, n (%) | 4067 (66.51%) | 851 (69.36%) |  |  |
| COPD, n (%) | 753 (12.3%) | 136 (11.1%) | 0.247 | 0.038 |
| Hypertension, n (%) | 3417 (55.9%) | 645 (52.6%) | 0.038 | 0.067 |
| Diabete, n (%) | 1838 (30.1%) | 405 (33.0%) | 0.048 | 0.064 |
| CKD, n (%) | 1836 (30%) | 331 (27.0%) | 0.036 | 0.068 |
| SOFA, mean±SD | 4.56±3.24 | 4.87±3.65 | 0.003 | 0.09 |

**Supplementary Table 3. Coefficient Values of LASSO Regression.**

| Supplementary Table 3. Coefficient Values of LASSO Regression | |
| --- | --- |
| Features | Coefficient Value |
| Age | 0.002337313 |
| Gender | 0 |
| BMI | 0 |
| Race | 0 |
| Los_hospital | 0 |
| Los_icu | 0.244637157 |
| Tg | 0 |
| Glucose | 0.000604581 |
| Sodium | 0.010962664 |
| Creatinine | 0.147496746 |
| BUN | 0.002271985 |
| Albumin | 0 |
| INR | 0 |
| PT | 0 |
| PTT | 0 |
| ALT | 0 |
| AST | 4.93743E-05 |
| ALP | -0.001303456 |
| CK_MB | 0 |
| Heart Rate | -6.12492E-05 |
| SBP | -0.00223881 |
| DBP | 0 |
| Calcium | 0 |
| Chloride | 0 |
| Potassium | 0 |
| Bicarbonate | 0 |
| PCI | 0 |
| WBC | 0 |
| RBC | 0 |
| Hemoglobin | 0.000624346 |
| Platelet | 0 |
| SAPSII | 0 |
| SOFA | 0.108980996 |
| SIRS | 0 |
| APSII | 0 |
| OASIS | 0.023629624 |
| LODS | 0.091242693 |
| CKD | 0.166536565 |
| RRT | 0 |
| Bypass | 0.072034512 |
| COPD | 0 |
| Hypertension | 0.02790116 |
| Diabete | 0 |
| TyG | 0.090966635 |

**Supplementary Table 4. Comparison of model performance with and without inclusion of ICU length of stay (LOS_ICU) across all cohorts.**

| Supplementary Table 4. Comparison of model performance with and without inclusion of ICU length of stay (LOS_ICU) across all cohorts. | | | | | |
| --- | --- | --- | --- | --- | --- |
|  | Model include LOS_ICU | | Model exclude LOS_ICU | | p value |
| cohorts | AUC | CI | AUC | CI |  |
| Training | 0.851 | 0.825, 0.878 | 0.849 | 0.822, 0.876 | 0.909 |
| Internal validation | 0.831 | 0.786, 0.876 | 0.827 | 0.781, 0.872 | 0.896 |
| External validation | 0.813 | 0.790, 0.836 | 0.806 | 0.783, 0.829 | 0.087 |

**Supplementary Table 5. Clinical Variables and Corresponding Points System.**

| Variable | Value Range | Points | score |
| --- | --- | --- | --- |
| TyG index | 7 | 0 |  |
|  | 8 | 12 |  |
|  | 9 | 21 |  |
|  | 10 | 32 |  |
|  | 11 | 43 |  |
|  | 12 | 54 |  |
| BUN | 0 | 0 |  |
|  | 20 | 6 |  |
|  | 40 | 11 |  |
|  | 60 | 17 |  |
|  | 80 | 21 |  |
|  | 100 | 28 |  |
|  | 120 | 32 |  |
|  | 140 | 38 |  |
| SOFA | 0 | 0 |  |
|  | 2 | 11 |  |
|  | 4 | 22 |  |
|  | 6 | 32 |  |
|  | 8 | 43 |  |
|  | 10 | 55 |  |
|  | 12 | 66 |  |
|  | 14 | 77 |  |
|  | 16 | 88 |  |
|  | 18 | 100 |  |
| Age | 20 | 0 |  |
|  | 40 | 5 |  |
|  | 60 | 8 |  |
|  | 80 | 12 |  |
| Albumin | 5 | 0 |  |
|  | 4.5 | 8 |  |
|  | 4 | 18 |  |
|  | 3.5 | 28 |  |
|  | 3 | 37 |  |
|  | 2.5 | 44 |  |
|  | 2 | 54 |  |
|  | 1.5 | 65 |  |
|  | 1 | 74 |  |
| Sodium | 105 | 0 |  |
|  | 115 | 8 |  |
|  | 125 | 14 |  |
|  | 135 | 22 |  |
|  | 145 | 39 |  |
|  | 155 | 37 |  |
| SBP | 180 | 0 |  |
|  | 140 | 5 |  |
|  | 100 | 11 |  |
|  | 60 | 17 |  |

**Supplementary Table 6. Comparison of model performance before and after exclusion of patients who died prior to AKI onset.**

| Supplementary Table 6. Comparison of model performance before and after exclusion of patients who died prior to AKI onset. | | | |
| --- | --- | --- | --- |
| Cohort | AUC | 95% CI | p-value |
| Training cohort | 0.854 | 0.827 – 0.880 | - |
| Adjusted cohort | 0.849 | 0.826 – 0.872 | 0.7921 |
| Adjusted cohort refers to the training cohort after excluding 12 patients who died prior to AKI onset. | | | |
